# Influence of Microbial Metabolites on the Nonspecific Permeability of Mitochondrial Membranes under Conditions of Acidosis and Loading with Calcium and Iron Ions

**DOI:** 10.3390/biomedicines9050558

**Published:** 2021-05-17

**Authors:** Nadezhda Fedotcheva, Andrei Olenin, Natalia Beloborodova

**Affiliations:** 1 Institute of Theoretical and Experimental Biophysics, Russian Academy of Sciences, Institutskaya Street 3, 142290 Pushchino, Russia; 2 V.I. Vernadsky Institute of Geochemistry and Analytical Chemistry, Russian Academy of Sciences, 19 Kosygin Street, 119991 Moscow, Russia; ayolenin@yandex.ru; 3 Federal Research and Clinical Center of Intensive Care Medicine and Rehabilitology, 25-2 Petrovka Street, 107031 Moscow, Russia; nvbeloborodova@yandex.ru

**Keywords:** mitochondrial dysfunction, MPTP, swelling, microbial metabolites, acetyl phosphate, sepsis, iron, acidosis

## Abstract

Mitochondrial dysfunction is currently considered one of the main causes of multiple organ failure in chronic inflammation and sepsis. The participation of microbial metabolites in disorders of bioenergetic processes in mitochondria has been revealed, but their influence on the mitochondrial membrane permeability has not yet been studied. We tested the influence of various groups of microbial metabolites, including indolic and phenolic acids, trimethylamine-N-oxide (TMAO) and acetyl phosphate (AcP), on the nonspecific permeability of mitochondrial membranes under conditions of acidosis, imbalance of calcium ions and excess free iron, which are inherent in sepsis. Changes in the parameters of the calcium-induced opening of the mitochondrial permeability transition pore (MPTP) and iron-activated swelling of rat liver mitochondria were evaluated. The most active metabolites were indole-3-carboxylic acid (ICA) and benzoic acid (BA), which activated MPTP opening and swelling under all conditions. AcP showed the opposite effect on the induction of MPTP opening, increasing the threshold concentration of calcium by 1.5 times, while TMAO activated swelling only under acidification. All the redox-dependent effects of metabolites were suppressed by the lipid radical scavenger butyl-hydroxytoluene (BHT), which indicates the participation of these microbial metabolites in the activation of membrane lipid peroxidation. Thus, microbial metabolites can directly affect the nonspecific permeability of mitochondrial membranes, if conditions of acidosis, an imbalance of calcium ions and an excess of free iron are created in the pathological state.

## 1. Introduction

Mitochondria play a key role in cellular metabolism, survival and homeostasis by participating in energy production, intracellular signaling and cell death regulation. Dysfunction of mitochondria has been associated with several pathologies, including neurodegenerative diseases, cancer, cardiovascular disorders, inflammation and other. Mitochondrial dysfunction is of special interest in the case of the development of a life-threatening pathological process associated with an inadequate response of the body to excessive bacterial load, which is called “sepsis”. The progression of multiple organ failure in sepsis is inevitably accompanied by a high frequency of deaths; at the same time, studies have confirmed the important role of mitochondrial disorders in the violation of the function of organs and systems in sepsis, which remains a keen interest in the search for mechanisms of this process [1,2]. Bioenergetic failure, up to metabolic reprogramming, decreased activity of some mitochondrial enzymes and respiratory chain complexes are important pathophysiological mechanisms underlying multiple organ failure, and the appearance of signs of mitochondrial dysfunction and multiple organ failure depends on the stage of the inflammatory process [3,4]. Multiple organ failure in early sepsis is mainly functional, not accompanied by tissue destruction, necrosis or other irreversible structural damage. The potential reversibility of disorders, the possibility of restoring organ function under the condition of functional rehabilitation of mitochondria, encourages the search for new mechanisms or factors involved in the development of mitochondrial-mediated cellular dysfunction and immunosuppression [5].

Along with oxidative stress, altered nitric oxide production and other mechanisms leading to microcirculatory and endothelial dysfunction, the permeability of the tissues and intestinal barriers increases, which is accompanied by an excessive intake of microbial metabolic products into the blood, not only from the focus of infection but also from the gut. As is known, trillions of bacteria live and function in the human body, which are united by the term “microbiota”. During critical illness, particularly in sepsis, the taxonomic diversity of the microbiota is disrupted, which affects the qualitative and quantitative composition of its metabolites [6].

Numerous human microbiota produce metabolites that may enter the bloodstream and exert systemic effects on various bodily functions. Microbial metabolites are involved in the regulation of the immune system [7], the central nervous system [8], metabolism and epigenetic control [9,10]. 

Although microbial metabolites are essential for the normal metabolism and homeostasis of the body, some of them can be toxic at high concentrations [11,12,13]. 

Among microbial metabolites, several groups of compounds have been identified that affect the functions of mitochondria. These include short-chain fatty acids, trimethylamine and its derivatives, indole and phenolic derivatives of aromatic amino acids and some other metabolites of microbial origin [14]. Short-chain fatty acids are predominantly incorporated into mitochondrial metabolism or act indirectly as signaling molecules [15]. The trimethylamine derivative trimethylamine-N-oxide (TMAO) has been shown to reduce the oxidation of pyruvate and fatty acids in mitochondria [16,17]. Metabolites from the group of indolic and phenolic derivatives inhibit mitochondrial dehydrogenases [4,18] and affect the production of reactive oxygen species (ROS) [19,20]; some of them can influence the permeability of mitochondrial and cell membranes [14]. A study of the role of microbial metabolites in the regulation of mitochondrial functions is especially important due to the fact that mitochondrial dysfunction has recently been considered as one of the main causes of multiple organ failure in chronic inflammation and sepsis. Since mitochondria play a key role in many cellular processes, such as calcium homeostasis and the production of ROS, and are the key regulators of cell death, they are often considered a potential therapeutic target in sepsis [21,22].

Previously, the influence of phenolic acids of microbial origin on the main functions of mitochondria respiration, membrane potential and ROS production was revealed [23]. Under conditions of acidosis and a deficiency of the oxidation substrate, which, as a rule, accompany the development of sepsis, their effect on mitochondria increased significantly [24]. Moreover, acidosis correlated with a rise in the concentrations of phenolic acids and mitochondrial metabolites in the blood of patients at different stages of sepsis [4]. In addition to acidosis and substrate deficiency, another factor characteristic of sepsis and essential for mitochondrial functions is an increase in the level of free iron in the blood and tissues. Recently, it has been shown that a higher blood iron level in sepsis correlates with increased mortality [25]. It is known that an excess of iron ions accumulates mainly in mitochondria, where it induces lipid peroxidation of mitochondrial membranes and, as a consequence, disruption of a number of bioenergetic processes [26,27]. Currently, there are data on the participation of microbial metabolites in the regulation of iron homeostasis [28,29] and the possible role of some of them, namely phenolic acids, in the binding of iron ions [30]. Additionally, there is evidence indicating that indole derivatives indole-3-propionic acid and 5-hydroxy-indole-3-acetic acid in the concentration range of 0.25-4.0 mM can act as inhibitors of lipid peroxidation induced by iron ions [31]. The same relates to disorders of calcium homeostasis in infections and sepsis. Changes in Ca^2+^ levels in bodily fluids and subcellular compartments were found in different sepsis models. They were manifested in a decrease in the blood Ca^2+^ concentration and calcium overload in cytosol, endoplasmic reticulum and mitochondria [32,33,34]. Moreover, it has been shown that the inhibition of mitochondrial permeability transition by cyclosporine A prevented sepsis-induced myocardial dysfunction and mortality [35]. According to some data, acidosis acts on the mitochondrial permeability in the same direction as calcium and iron overload. As has been shown, extracellular acidification induced mitochondrial functional impairment and MPTP opening, while co-treatment with cyclosporine-A prevented these disorders [36,37]. 

The participation of microbial metabolites in the modulation of the mitochondrial membrane permeability, especially in conditions of acidosis and overload of calcium and iron ions, has not yet been studied. In this work, we investigated the influence of microbial metabolites of various groups, including indolic and phenolic acids, TMAO and acetyl phosphate (AcP), on the nonspecific permeability of mitochondrial membranes activated by calcium and iron ions as well as by acidification of medium. Changes in the parameters of the calcium-induced opening of the mitochondrial permeability transition pore (MPTP) and iron-activated swelling of mitochondria were measured in a series of experiments on isolated rat liver mitochondria. We found that the influence of these metabolites on the permeability of mitochondrial membranes was significantly enhanced under these conditions and was prevented by the lipid radical scavenger butyl-hydroxytoluene (BHT). 

## 2. Materials and Methods

### 2.1. Reagents and Chemicals

Benzoic acid (BA, ≥99.5%), phenyllactic acid (PLA, ≥98%) and 4-hydroxyphenyllactic acid (HPLA, ≥97%) were obtained from Merck (Darmstadt, Germany); all other reagents were from the Sigma–Aldrich Corporation (St. Louis, MO, USA). 

### 2.2. Preparation of Rat Liver Mitochondria

Mitochondria were isolated from adult Wistar male rats. The study was conducted in accordance with the ethical principles formulated in the Helsinki Declaration on the care and use of laboratory animals. Manipulations were carried out by the certified staff of the Animal Department of the Institute of Theoretical and Experimental Biophysics (Russian Academy of Sciences and approved by the Commission on Biomedical Ethics of ITEB RAS (N28/2021, 9 February 2021)). During the study, the animals were kept in wire-mesh cages at room temperature (22 °C) with a light/dark cycle of 12 h. Mitochondria from the liver of anesthetized animals were isolated using the standard method [14]. The liver was rapidly removed and homogenized in an ice-cold isolation buffer containing 300 mM sucrose, 1 mM EGTA and 10 mM HEPES–Tris (pH 7.4). The homogenate was centrifuged at 600× *g* for 7 min at 4 °C, and the supernatant fraction was then centrifuged at 9000× *g* for 10 min to obtain mitochondria. Mitochondria were washed twice in the above medium without EGTA and BSA. The final mitochondrial pellet was suspended in the washing medium to yield 60 mg protein/mL and kept on ice for analysis. Mitochondria with a respiratory control ratio of around 4 were used throughout all experiments.

### 2.3. Determination of the Ca^2+^-Induced MPTP Opening

The opening of the MPTP was registered as a steep rise in calcium ions in the incubation medium and dissipation of the mitochondrial membrane potential. MPTP opening was induced by the sequential loading of the incubation medium with CaCl_2_. The difference in the electric potential on the inner mitochondrial membrane was measured from the redistribution of lipophilic cation tetraphenylphosphonium (TPP^+^) between incubation medium and mitochondria. The concentration of TPP^+^ in the incubation medium was recorded by a TPP^+^-selective electrode (Nico, Moscow, Russia). Changes in the calcium ion concentration in the incubation medium were recorded by a Ca^2+^-selective electrode (Nico, Moscow, Russia). The concentration of TTP^+^ and Ca^2+^ was registered simultaneously in an open chamber of volume 1 mL containing 1.0–1.2 mg mitochondrial protein under continuous stirring. MPTP opening was induced by the sequential loading of the incubation medium with 25 µM Ca^2+^ (CaCl_2_). The mitochondrial calcium retention capacity (CRC) was determined as the total concentration of added Ca^2+^ required for pore opening. The incubation medium contained 125 mM KCl, 15 mM HEPES, 1.5 mM phosphate, 5 mM succinate, pH 7.4. All microbial metabolites tested are hydrophilic and completely soluble in water when the pH value is adjusted to neutral. 

### 2.4. Determination of Mitochondrial Swelling

The swelling of mitochondria was measured at a wavelength of 540 nm using an Ocean Optics USB4000 spectrophotometer. Swelling was assessed by measuring the changes in optical density during incubation. Mitochondria at a concentration of mitochondrial protein of 0.3–0.4 mg/mL were incubated in the same buffer (125 mM KCl, 15 mM HEPES, 1.5 mM phosphate, 5 mM succinate). Swelling was stimulated by the addition of 50–100 µM FeSO_4_ × 7H_2_O or medium acidification to pH 6.7. The influence of microbial metabolites on mitochondrial swelling was assessed by changes in the lag phase preceding the swelling and the rate of the decrease in optical density. 

### 2.5. Statistical Analysis

The data given represent the means ± standard error of means (SEM) from five to seven experiments or are the typical traces of three to five identical experiments with the use of different mitochondrial preparations. The statistical significance was estimated by the Student’s *t*-test with *p* < 0.05 as the criterion of significance.

## 3. Results

### 3.1. The Influence of Indolic and Phenolic Acids on the Induction of Calcium-Dependent MPTP

**Figure 1:**
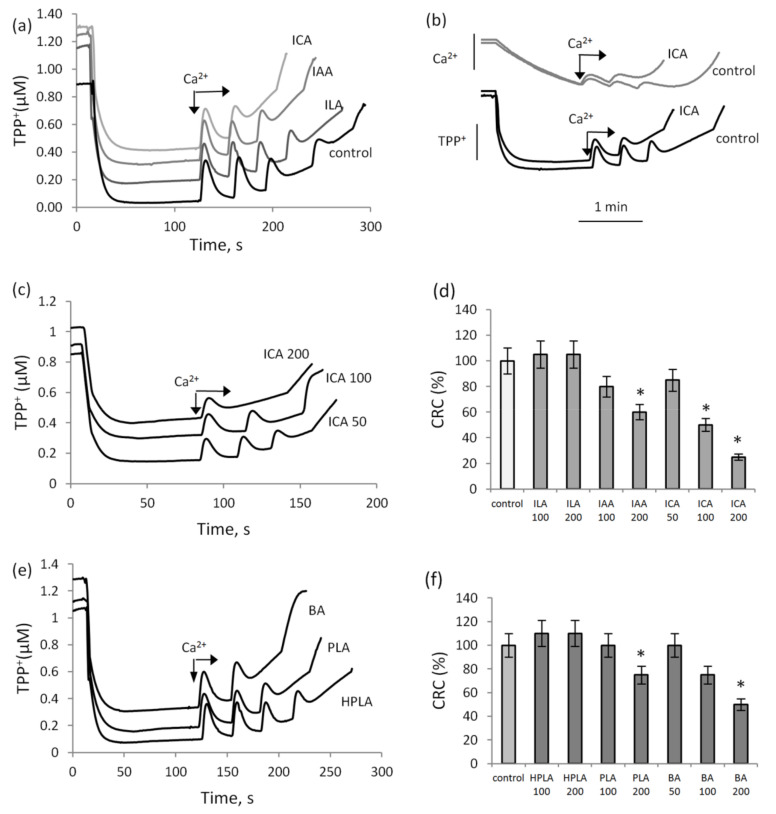
Influence of indolic and phenolic acids on MPTP opening induced by calcium ions. MPTP opening in the course of successive additions of 25 µM CaCl_2_ in the presence of indolic acids at a concentration of 100 µM, determined by a drop in the membrane potential (**a**) and by Ca^2+^ release (**b**) in the control and in the presence of indole carboxylic acid (ICA, 100 µM). A decrease in the calcium retention capacity (CRC) with an increase in ICA concentration from 50 to 200 µM (**c**) and changes in the CRC at different concentrations of other indolic acids (**d**). MPTP opening measured by the membrane potential changes in the course of successive additions of 25 µM CaCl_2_ in the presence of phenolic acids at a concentration of 100 µM (**e**) and changes in the CRC at different concentrations of phenolic acids (**f**). Asterisk (*) indicates values that differ significantly from the control values (*p* < 0.05).

In these experiments, the threshold concentrations of calcium ions required for pore opening in the presence of indolic and phenolic acids in comparison with the control were estimated. Figure 1 shows the effect of indole derivatives—indolcarboxylic acid (ICA), indolacetic acid (IAA) and indollactic acid (ILA)—on the membrane potential and the accumulation of calcium ions in mitochondria in the course of successive 25 µM CaCl_2_ supplementations. Indolic acids, with the exception of ILA, reduced the threshold calcium ion concentration inducing MPTP opening. ICA at a concentration of 200 µM decreased the CRC by almost three times; IAA at the same concentration decreased the CRC by 30%, while ILA had no effect on MPTP induction. MPTP opening was accompanied by a decrease in the membrane potential and a release of calcium ions from mitochondria, as shown in the experiment with the addition of ICA (Figure 1b). The activating effect of ICA on MPTP opening rose with an increase in its concentration from 50 to 200 µM (Figure 1c). ICA began to activate MPTP opening at a concentration of 50 µM, IAA at a concentration of 100 µM, and ILA had no effect in the entire concentration range (Figure 1d). Figure 1e,f show the influence of another group of aromatic microbial metabolites, phenolic acids, on calcium ion-induced MPTP opening. Benzoic acid (BA) at a concentration of 100 µM and phenyllactic acid (PLA) at a concentration of 200 µM decreased the CRC by two times and by 25%, respectively, while hydroxyphenyllactic acid (HPLA) slightly increased the threshold calcium concentration required for MPTP opening. Thus, ICA and BA activated MPTP opening to a greater extent than the other indolic and phenolic acids.

### 3.2. The Influence of Indolic and Phenolic Acids on the Nonspecific Permeability of Mitochondrial Membranes in the Presence of Iron Ions

**Figure 2:**
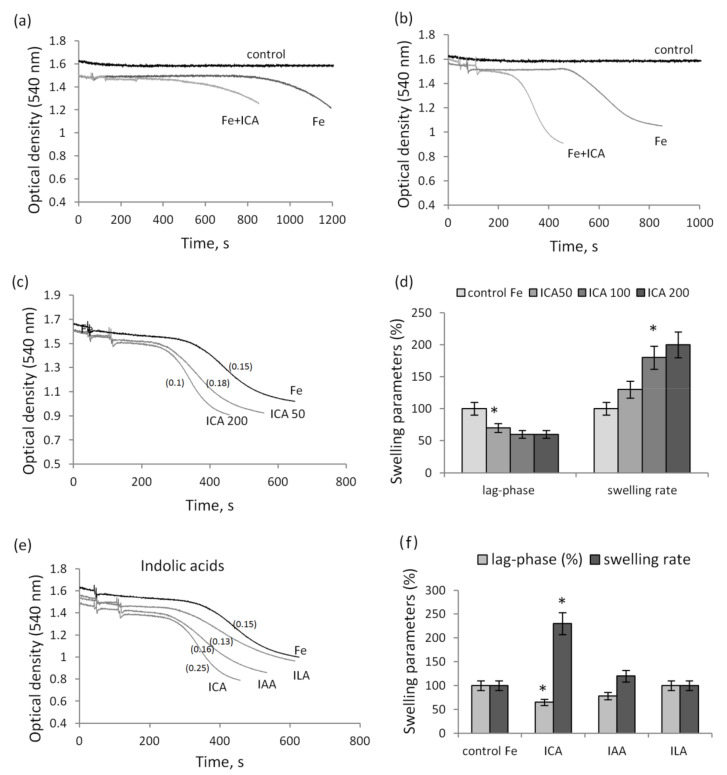
Influence of indolic acids on the mitochondrial swelling activated by iron ions. Activation of swelling during incubation with iron ions (50 µM FeSO_4_) and 100 µM ICA (**a**, **b**) and at different ICA concentrations (**c**); changes in the swelling parameters in the presence of different ICA concentrations (**d**). The influence of indolic acids on swelling (**e**) and the swelling parameters: the lag phase and the swelling rate (**f**). The swelling rate (Δ/min) is indicated in parentheses. Asterisk (*) indicates values that differ significantly from the control values (*p* < 0.05).

Long-term incubation with iron ions is accompanied by the activation of the swelling of mitochondria. As shown in Figure 2a,b, ferrous ions induced the swelling with a lag phase that varied between 6 and 12 min in different mitochondrial preparations. In all cases, the addition of indolic or phenolic acids activated swelling, which manifested itself either as a decrease in the lag phase, or as an increase in the swelling rate, or changes in both parameters together. The degree of activation was different for each metabolite and depended on its concentration. ICA decreased the lag phase and activated the swelling rate by 20% at a concentration of 50 µM and almost two times at a concentration of 200 µM (Figure 2c). Furthermore, with an increase in the ICA concentration, an increase in the swelling rate occurred more intensively than a decrease in the lag phase (Figure 2d). The other indolic acids, IAA and ILA, in the same concentration range only slightly reduced the lag phase and did not activate the swelling rate (Figure 2e,f).

**Figure 3:**
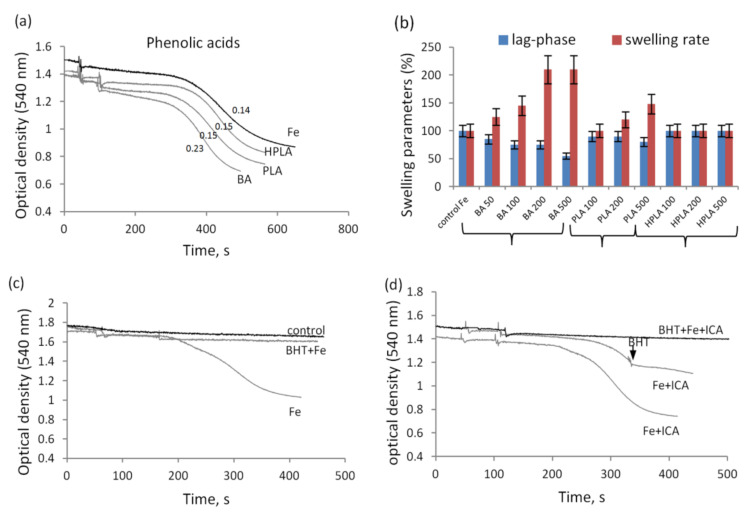
Influence of phenolic acids on mitochondrial swelling induced by iron ions and its elimination by BHT. Activation of the swelling of mitochondria during incubation with iron ions (50 µM FeSO_4_) and phenolic acids at a concentration of 100 µM (**a**); changes in the swelling parameters in the presence of 50 µM FeSO_4_ and phenolic acids in the concentration range 100–500 µM (**b**); and the elimination by BHT of swelling induced by 50 µM FeSO_4_ alone (**c**) and with 100 µM ICA (**d**).

In the presence of iron ions, phenolic acids produced similar effects. At the same concentration (100 µM), BA activated, and HPLA and HPLA had only an insignificant effect on, iron-induced swelling of mitochondria (Figure 3a). With an increase in concentration to 200 µM, BA doubled the swelling rate, PLA activated swelling by 15% and 50% at concentrations of 200 and 500 µM, respectively, and HPLA had no effect in the entire concentration range from 100 to 500 µM (Figure 3b). Additionally, the increase in the swelling rate was more pronounced than the decrease in the lag phase.

### 3.3. The Influence of Indolic and Phenolic Acids on the Permeability of Mitochondrial Membranes under Conditions of Acidosis

**Figure 4:**
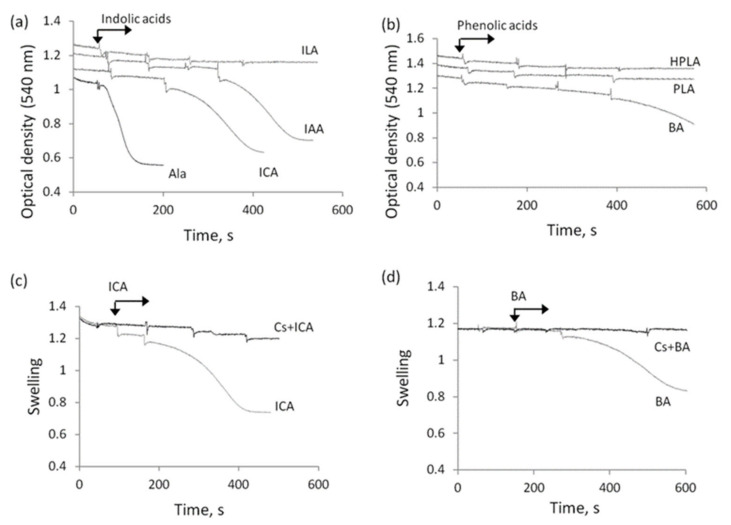
Influence of an excess of indolic and phenolic acids on mitochondrial swelling at neutral pH. Activation of the swelling in the course of successive additions of indolic (**a**) and phenolic (**b**) acids at a concentration of 100 µM each; the inhibition by CsA of swelling induced by CA (**c**) and BA (**d**).

Another factor linking mitochondrial dysfunction and sepsis is acidosis. Figure 4 shows the effect of indolic and phenolic acids on the swelling of mitochondria under acidification of the incubation medium. Their effect on the swelling at neutral pH was also studied. In these experiments, the possible induction of mitochondrial swelling was examined in the course of successive additions of acids at a concentration of 200 µM each (Figure 4a). The swelling induced by alamethicin, a peptide that forms nonspecific voltage-dependent ion channels, served as a control parameter. Alamethicin induced rapid and high-amplitude swelling. At neutral (pH 7.4), ICA and IAA induced swelling at concentrations of 250 and 500 µM, respectively, while ILA had no effect even at concentrations above 500 µM. Phenolic acids had a similar effect (Figure 4b). BA induced mitochondrial swelling at a concentration of around 250 µM, and PLA and HPLA had no effect even at concentrations above 500 µM. ICA- and BA-induced swelling was prevented by cyclosporine A (Figure 4c,d).

**Figure 5:**
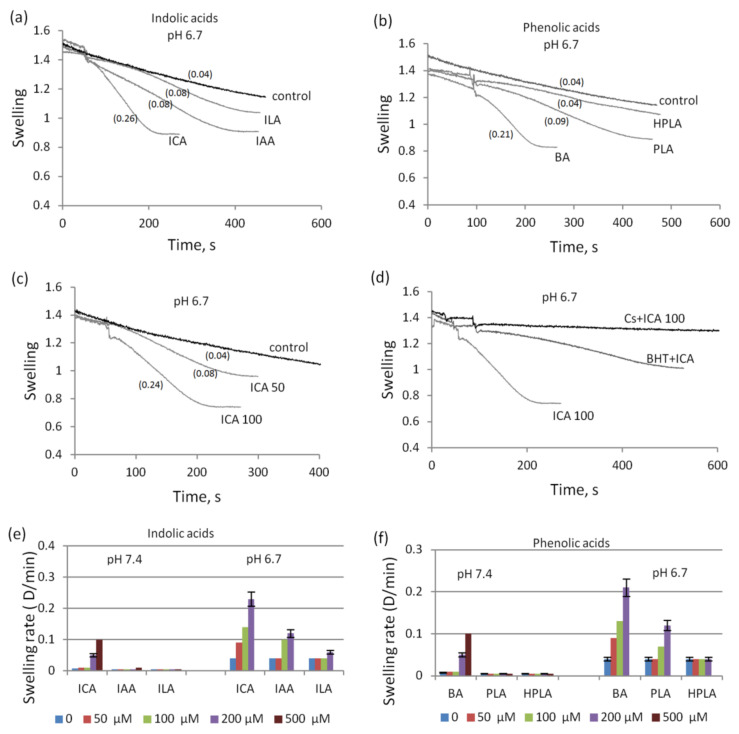
Influence of indolic and phenolic acids on mitochondrial swelling activated by acidification. Activation of swelling by indolic (**a**) and phenolic (**b**) acids at a concentration of 100 µM each during incubation at pH 6.7; swelling in the presence of 50 and 100 µM ICA (**c**) and its inhibition by CsA and BHT (**d**); comparison of the swelling rate in the presence of indolic (**e**) and phenolic (**f**) acids at different concentrations (50–500 µM) at pH 7.4 and 6.7. The swelling rate (Δ/min) is indicated in parentheses.

With the acidification of the incubation medium (pH 6.7), the effect of indolic and phenolic acids on the swelling of mitochondria greatly increased (Figure 5a,b). ICA and BA stimulated swelling at significantly lower concentrations than at neutral pH. Whereas at neutral pH ICA stimulated swelling at a concentration of 200 µM, at pH 6.7, it acted even at a concentration as low as 50 µM (Figure 5c). Furthermore, as their concentration was increased, not only the rate but also the amplitude of swelling increased (Figure 5c). The swelling induced by indolic and phenolic acids was inhibited by cyclosporine A and BHT, as shown by the example with ICA supplementation (Figure 5d). Upon acidification, all other indolic and phenolic acids weakly stimulated the swelling in concentrations ranging from 100 to 500 µM (Figure 5e,f).

### 3.4. The Influence of Acetyl Phosphate and TMAO on the Nonspecific Permeability of Mitochondrial Membranes

**Figure 6:**
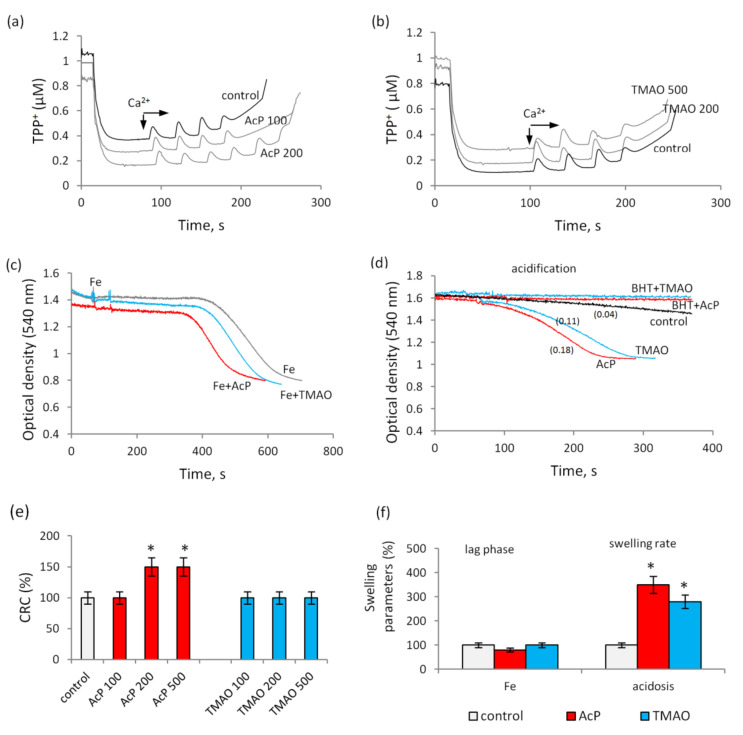
Influence of TMAO and AcP on the MPTP opening induced by calcium ions and on swelling activated by iron ions and medium acidification. MPTP opening in the course of successive additions of 25 µM CaCl_2_ in the presence of AcP (**a**) and TMAO (**b**) at indicated concentrations; iron-activated swelling in the presence of AcP and TMAO at concentrations of 200 µM (**c**); swelling activated by acidification (pH 6.7) in the presence of AcP and TMAO at a concentration of 100 µM and its elimination by BHT, the swelling rate (Δ/min) is indicated in parentheses (**d**); calcium retention capacity at different AcP and TMAO concentrations (100–500 µM) (**e**) and swelling parameters in the presence of AcP and TMAO at concentrations of 100 µM under iron loading and medium acidification (**f**). Asterisk (*) indicates values that differ significantly from the control values (*p* < 0.05).

The main indicators of the influence of indole and phenolic acids on the permeability of mitochondrial membranes were studied for two other microbial metabolites, acetyl phosphate (AcP) and TMAO. Figure 6 shows the effect of these metabolites on MPTP opening induced by calcium, as well as on the mitochondrial swelling activated by iron and acidification. In contrast to indolic and phenolic acids, AcP had an opposite, protective effect, increasing the threshold concentration of calcium ions required for MPTP opening. At a concentration of 200 µM, AcP increased the CPC 1.5 times (Figure 6a). A further increase in its concentration to 500 µM did not enhance the effect. On the contrary, TMAO at concentrations from 100 to 500 µM did not affect the Ca-induced opening of MPTP (Figure 6b).

Both metabolites had insignificant effects on iron-activated mitochondrial swelling. Of these compounds, only AcP insignificantly reduced the lag phase without affecting the swelling rate (Figure 6c). Under acidification, both metabolites at a concentration of 100 µM activated swelling, which was completely inhibited by BHT (Figure 6d). 

## 4. Discussion 

This study identified microbial metabolites that have an impact on the nonspecific permeability of mitochondrial membranes, as well as the conditions that activate their influence. The most active metabolites are ICA and BA, which represent, respectively, the groups of indolic and phenolic acids. Both metabolites reduced the threshold calcium ion concentration required for MPTP opening and activated mitochondrial swelling induced by iron ions and acidification. At high concentrations, they stimulated swelling even in the absence of additional conditions. The other metabolites from the groups of indole and phenolic acids—IAA, ILA, PLA—were less active, and HPLA did not activate MPTP opening and mitochondrial swelling under all incubation conditions. These metabolites can be arranged in decreasing order of their influence on mitochondria as follows: ICA ≥ BA > IAA > ILA ≥ PLA > HPLA. Active metabolites can also include AcP, which exerted an opposite, protective effect on the induction of MPTP by calcium ions, increasing the threshold calcium concentration 1.5 times. A representative of another group of microbial metabolites, TMAO, did not affect the MPTP opening even at high concentrations. The conditions that activate the nonspecific permeability of mitochondrial membranes were selected in accordance with those observed in infections and sepsis. These include disturbances in the homeostasis of calcium and iron, as well as acidosis. Moreover, what is important is that these processes are functionally related. It was previously shown that the mitochondrial permeability transition plays a critical role in sepsis-induced multiple organ dysfunction and increased mortality rate, which were reduced by cyclosporine, an inhibitor of MPTP [35]. Calcium overload is considered to be the main trigger of the opening of MPTP, although its state also depends on other concomitant factors, such as oxidative stress, depletion of adenine nucleotides and mitochondrial depolarization. The consequence of MPTP opening is the loss of the membrane potential, inhibition of respiration and ATP synthesis, swelling of mitochondria and rupture of outer membrane, which leads to the cell death. It has been shown that acidosis promotes MPTP opening [36,37]. Additionally, acidosis provoked the release of iron ions from various cellular sources, including endosomes and lysosomes, as well as from weakly bound forms with anions such as citrate and nucleotides [26]. In these cases, the concentration of free iron can increase up to 300 µM, and, importantly, all excess iron accumulates mainly in mitochondria. As follows from our data, both factors, acidosis and iron load, led to an increase in the nonspecific permeability of mitochondrial membranes. The role of microbial metabolites in these processes was first shown in our study. 

Based on the evidence for the possible interaction of phenolic derivatives with iron ions, it may be assumed that metabolites from the group of phenolic acids are able to reduce the effect of iron on mitochondria. There are several types of reactions of phenolic compounds with iron [38,39,40,41]. First, there are redox reactions with the participation of iron ions, where phenol-containing acids can act as reducing agents. In this case, as follows from Equation (1), the most probable direction is the oxidation of the phenol fragment to the corresponding quinone:

HOOC-R-C_6_H_4_-OH + Fe^3+^ → HOOC-R = C_6_H_4_ = O + Fe^2+^ (1)

The formation of a complex with Fe^2+^ can occur according to the following Equations (2) and (3):

Fe^2+^ + R-O^−^ = [Fe-O-R]^+^ (2)

Fe^2+^ + R-OH = [Fe-O-R]^+^ + H^+^ (3)

If the main chain of the phenolic acid contains an alcohol group, then it can be transformed to a carbonyl, wherein phenolic hydroxyls are oxidized more easily than alcohol. Second, in addition to redox, there can be acid-base interactions. For iron salts, as Equation (4) shows, hydrolysis can occur with the formation of basic salts:

Fe^2+^ + H_2_O = FeOH^+^ + H^+^ (4)

Reactions of the same type with the participation of phenolic acids generally occur as follows from Equation (5):

R-O^−^ + H_2_O = R-OH + OH^−^ (5)

In general, the equilibrium takes place according to the reaction represented by Equation (6):

R-COOH + Fe^2+^ = R-COOFe^+^ + H^+^ (6)

The probability of the first or second type is determined by the pH of the medium. Indeed, both types of interactions of phenolic compounds with iron have been confirmed by experimental data. Phenolic acids containing hydroxyl groups in the phenolic ring have been shown to bind iron ions to form complexes in the 3:1 ratio, which suggests that they can act as iron chelators [30]. It was also shown that Fe(II) is mainly adsorbed onto carboxylic groups at neutral pH, whereas phenolic groups play a major role at basic pH values [42].

In our experiments, redox reactions can be carried out with the participation of those metabolites that contain hydroxyl groups. These include HPLA, PLA and ILA. It should be noted that nitrogen-containing heterocycles, such as indole and its derivatives, are much more stable in oxidation reactions in comparison with alcohols (phenols). Iron ions form complexes with them rather than entering into redox reactions. Complexes of this type with porphyrin are stable and exist in heme. The second type of reaction with iron, acid-base interactions with the participation of carboxyl groups, can occur with all indole and phenolic acids. 

Despite our assumptions, HPLA, PLA and ILA had no protective effect on the iron-induced activation of membrane permeability determined by swelling of mitochondria. However, unlike other phenolic and indolic acids, they did not enhance the effect of iron on the rate and amplitude of mitochondrial swelling. Probably, this is due to the fact that either the complexes with iron are unstable, or the complexation occurs under more specific conditions, which requires further research. On the other hand, since the effects of iron are associated with the activation of lipid peroxidation, it can be assumed that the antioxidant properties of hydroxyl-containing metabolites and pro-oxidant properties of other phenolic derivatives, which have been previously revealed in our studies, play a role in this process [20]. 

Acidosis turned out to be the most powerful factor mediating and intensifying the effect of microbial metabolites on the nonspecific permeability of mitochondrial membranes. Under these conditions, all microbial metabolites, with the exception of HPLA, activated mitochondrial swelling at lower concentrations than when loaded with calcium or iron ions. All the effects of microbial metabolites, both under iron loading and in acidosis, were completely eliminated by BHT, an inhibitor of lipid peroxidation, indicating a general pathway by which these factors influence the permeability of mitochondrial membranes. BHT is a phenolic radical-trapping antioxidant which blocks lipids autoxidation by converting peroxyl radicals to non-radical products [43]. Based on these data, it can be assumed that microbial metabolites activate mitochondrial swelling, enhancing lipid peroxidation, which is in good agreement with the data showing that lipid peroxidation is enhanced with decreasing extracellular pH and loading with free iron ions [44,45]. Furthermore, iron-activated lipid peroxidation has been shown to sensitize MPTP opening by calcium ions [27].

As for TMAO, its effect on mitochondria can also be accomplished via redox-dependent pathways. TMAO is known to significantly trigger oxidative stress, activate inflammasome and inhibit endothelial nitric oxide synthase [46]. In inflammatory processes, elevated TMAO levels lead to increased expression of pro-inflammatory cytokines and decreased expression of anti-inflammatory cytokines [47]. With regard to the relationship between TMAO and mitochondrial dysfunctions, of interest are the data showing a coupled increase in the level of TMAO, citrate and 2-oxoglutarate in urinary metabolomic profile in model experiments in rats with active or relapsing vasculitis [48]. In addition, TMAO promoted endothelial cell pyroptosis, a programmed cell death characterized by plasma membrane rupture via mitochondrial ROS production induced by SDH upregulation [49]. According to our data, TMAO stimulated mitochondrial swelling only under acidification and did not affect either calcium-induced MPTP opening or iron-induced swelling. Evidently, TMAO is less involved in redox reactions than indolic and phenolic acids. 

Unlike the above metabolites, AcP is not involved in redox reactions. As a prebiotic equivalent of acetyl-CoA, AcP is one of the most effective donors of acetyl groups in the reactions of protein acetylation, particularly non-enzymatic [50]. Currently, there are no data on the detection of AcP in humans; however, its very high concentration in bacterial cells suggests the possibility of its appearance in the host, especially during processes associated with bacteremia. For example, in *E. coli*, its concentration can reach 3 mM [51]. In mammalian mitochondria, AcP was detected only in one study as an intermediate of the pyruvate dehydrogenase complex, which forms in very low concentrations during the oxidation of pyruvate and rapidly degrades supposedly by acetyl phosphate hydrolase [52]. In our experiments, AcP acted as an inhibitor of Ca^2+^-induced MPTP, increasing the threshold concentration of calcium ions required for pore opening 1.5 times. It is important that the protective effect of AcP was observed only at neutral pH and disappeared with acidification of the incubation medium, which is in good agreement with the conditions for nonenzymatic acetylation, which, as is known, requires neutral or alkaline pH values [53]. Since acetylation affects such properties of proteins as activity, conformation, protein–protein interactions, stabilization and subcellular localization, it can be assumed that the effect of AcP is associated with the acetylation of some of MPTP components, which requires further clarification. 

The data obtained show an increase in the influence of microbial metabolites on the nonspecific permeability of mitochondrial membranes under acidification and loading with calcium and iron ions. All these factors lowered the effective concentrations of microbial metabolites several times. Of these, acidification was found to be the strongest factor. This is consistent with the view that acidosis is one of the main prognostic criteria of the severity of infections and sepsis. According to our data, acidosis correlated with mitochondrial dysfunction, which was assessed by an increase in the concentrations of mitochondrial metabolites in the blood of patients at different stages of sepsis [4]. Moreover, as mentioned above, acidosis promotes the transition of iron from bound forms to a free state, which in turn can intensify the development of the infection. It has been shown that a higher blood iron level in sepsis correlates with increased mortality [25]. Based on these data, it was proposed to use iron chelation as a potential therapy for polybacterial sepsis [54]. Indeed, iron binding by specific chelators was found to be effective in a number of cases of sepsis, and, moreover, iron chelation specifically altered the microbiota profile [55,56]. The same relates to disorders of calcium homeostasis in infections and sepsis, which are manifested in a decrease in Ca^2+^ concentration in the blood and its overload in cytosol and mitochondria [33,34,35]. As follows from our data, each of these factors enhances the effect of microbial metabolites on mitochondrial membrane permeability almost tenfold, reducing their active concentration. This is especially important in cases of infections associated with disorders of intestinal and cellular permeability. These data may be also useful in the context of the monitoring of the mitochondrial-dependent pathways of cell death via the calcium-induced apoptosis or iron-induced ferroptosis in severe pathological states, associated with infections and sepsis. 

**Figure 7:**
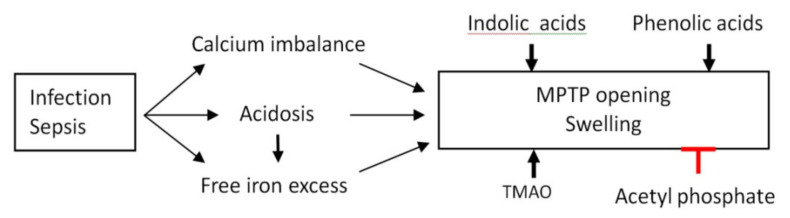
Contribution of microbial metabolites to the regulation of mitochondrial membrane permeability under conditions associated with infections and sepsis. The activation of the listed processes is shown with black arrows, the inhibition highlighted by a red sign.

Figure 7 summarizes the data regarding the contribution of microbial metabolites to the regulation of mitochondrial membrane permeability. As shown, infections and sepsis induce calcium imbalance and acidosis, which, in turn, promote the release of iron ions from bound forms. Microbial metabolites do not affect mitochondrial membrane permeability under physiological conditions but strongly activate MPTP opening and mitochondrial swelling under conditions of acidosis, calcium overload and an excess of free iron. Only acetyl phosphate showed a pronounced protective effect against the MPTP opening, probably via the nonenzymatic acetylation of MPTP components. 

It is important to note that acidosis and excess of calcium and iron ions significantly reduce the threshold concentrations of microbial metabolites, which affect nonspecific mitochondrial permeability. These concentrations of microbial metabolites are comparable to those found in humans in different physiological and pathological states. Thus, the total concentrations of phenolic acids were 404 µM in the colon in norm and 54.89 µM in serum in sepsis [23,57]. There is evidence indicating a strong increase in the level of phenylacetate up to 3.49 ± 0.33 mmol/l in the blood plasma of patients with end-stage renal disease [58]. The fivefold increase in the concentration of indolacetic acid (to 13.7 µM) in the blood was revealed during uremia [59]. Plasma TMAO levels in serum showed a large dynamic range from 0.06 to 312 µM after the consumption of specific trimethylamine-containing dietary nutrients, such as phosphatidylcholine, choline and carnitine [60]. 

## 5. Conclusions

Microbial metabolites participate in the regulation of the nonspecific permeability of mitochondrial membranes. Acidification and loading with calcium and iron ions significantly reduce the threshold concentrations of microbial metabolites that activate the MPTP opening and swelling of mitochondria. All the redox-dependent effects of metabolites were completely eliminated by BHT, a lipid radical scavenger, which indicates the activation of membrane lipid peroxidation by these metabolites. Thus, the overproduction of some microbial metabolites can directly affect the nonspecific permeability of mitochondrial membranes and the mitochondrial-dependent pathways of cell death, if conditions of acidosis, an imbalance of calcium ions and an excess of free iron are created in the pathological state.

## Data Availability

Not applicable.
